# Antimalarial, Antioxidant, and Toxicological Evaluation of Extracts of *Celtis africana*, *Grosseria vignei*, *Physalis micrantha*, and *Stachytarpheta angustifolia*

**DOI:** 10.1155/2021/9971857

**Published:** 2021-06-22

**Authors:** Michael Konney Laryea, Lawrence Sheringham Borquaye

**Affiliations:** ^1^Department of Chemistry, Kwame Nkrumah University of Science and Technology, Kumasi, Ghana; ^2^Central Laboratory, Kwame Nkrumah University of Science and Technology, Kumasi, Ghana

## Abstract

In many parts of the world, malaria undoubtedly poses a serious threat to health care systems. Malaria treatment has increasingly become complicated, primarily due to the emergence of widespread resistance of the malaria parasites to cheap and affordable malaria therapeutics. The use of herbal remedies to treat various ailments, including malaria and malaria-like ailments in Ghana is common. We herein report on the antiplasmodial and antioxidant activities as well as toxicological evaluation of four medicinal plants (*Celtis africana*, *Grosseria vignei*, *Physalis micrantha*, and *Stachytarpheta angustifolia)* commonly used to treat malaria in Ghana. Following Soxhlet extraction of plant samples in ethanol, extracts were screened against *Plasmodium falciparum* (3D7 strain) in an *in vitro* antiplasmodial assay. The phosphomolybdenum and DPPH (1, 1-diphenyl-2 picrylhydrazyl) assays were used to evaluate antioxidant activities while toxicity assessment was carried out in mice using the acute toxicity test and kidney and liver function tests. Extracts from *Celtis africana* and *Physalis micrantha* were very active towards the parasites with half-maximal inhibitory concentrations (IC_50_'s) of 29.1 and 3.5 *µ*g/mL, respectively. Extracts of *Grosseria vignei* and *Stachytarpheta angustifolia* were inactive, having IC_50_ values greater than 50 *µ*g/mL. All extracts exhibited excellent total antioxidant capacities (>800 mg/g AAE) and good DPPH radical scavenging potential (IC_50_ range of 300–900 *µ*g/mL). The median lethal dose (LD_50_) of all extracts in the toxicological evaluation was greater than 2000 mg/kg and there was no effect of extracts on the levels and activities of key biomarkers of liver and kidney function. The activities of these plants obtained in this study partly give credence to their folkloric use in herbal medicines and suggest that they could provide promising lead compounds for malaria drug discovery programs.

## 1. Introduction

In many countries in Sub-Saharan Africa, malaria remains a leading cause of mortality and morbidity among the populace, with detrimental effects on health care programs in these countries. The economic impact is disheartening. Despite numerous efforts and interventions put in place to combat the disease globally, the World Health Organization (WHO) reported in 2018 that malaria is still endemic in 80 countries and territories. This number has only been reduced from 108 since the year 2000 [1]. Various interventions have been developed for both vector and parasites to curb malaria infection in humans. The use of mosquito repellent sprays and creams and mosquito insecticide nets are some strategies commonly used to control the malaria vector [2]. Various drugs, either alone or in combination, have also been developed for use as curative or prophylactic interventions. Currently, artemisinin-based combination therapies are used for treating uncomplicated malaria in both children and adults [3]. The recent development of a malaria vaccine increases the artillery available to man in this war against malaria [4, 5]. However, reports of resistance to some drugs by the parasites have presented formidable challenges that need to be addressed for success [6, 7].

Oxidative stress has been suggested to play an important role in malaria pathology. Red blood cells infected with the *Plasmodium* parasite must eliminate reactive oxygen species (ROSs) produced during malaria infection to maintain cellular integrity. Large quantities of redox-active species are produced during metabolism in the fast-growing and multiplying parasite. These redox-active compounds are used by the parasite to break down host hemoglobin. Degradation of hemoglobin produces toxic free haem (ferri/ferroprotoporphyrin IX-FP) and ROSs, amongst others. A number of pathways exist for FP detoxification—sequestration into a crystalline haemozoin, FP degradation, reaction of FP with glutathione, and binding of FP to FP-binding proteins [8–11]. The presence of FP in the host cell could cause devastating redox damage to membranes and proteins in the host cell and lyse red blood cells [12]. Additionally, ROSs are produced by the host immune system during malaria infection and together contribute substantially to oxidative stress in the infected cell. The important role of oxidative stress in malaria pathology provides a promising target in antimalarial chemotherapy development [13]. The need to unearth newer, more potent therapeutic agents is great at the moment.

Traditional herbal preparations provide a useful avenue to prospect for therapeutic agents that are potent against the *Plasmodium* parasite. In Africa and many parts of Asia and the Americas, plant-based preparations are commonly used to treat various conditions, including malaria and malaria-like symptoms. The WHO estimates that over 70% of the population in Africa rely on herbal-based preparations for their health care needs [14]. A majority of compounds historically important in the fight against malaria have been derived from plants or modeled on structures derived from lead compounds isolated from plants. Examples of these include quinolone-based malaria drugs such as quinine and chloroquine as well as artemisinin and its derivatives [15]. The therapeutic success of these compounds has inspired the search for new antimalarial compounds from plant sources. A major challenge in the development of herbal medicine is the absence of toxicity data on the plant products administered [16]. Reports of adverse effects associated with the consumption of plant medicines necessitate a rigorous evaluation of the safety of potential plant products [17].

In Ghana, various medicinal plants are used in managing malaria and symptoms of malaria [18]. Many of these plants have not, however, been verified scientifically as possessing antiplasmodial activities. This work, therefore, seeks to validate the antiplasmodial properties of some plants commonly used to treat malaria in Ghana. The plants were selected from a survey of medicinal plants used in the country. Due to the important role of oxidative stress in malaria pathology, the antioxidant activities of the plants were also assessed. Finally, the effect of the plant extracts on some toxicological markers in a murine model was also investigated. We herein report on the antiplasmodial, antioxidant, and toxicological profile of the ethanolic extracts of *Celtis africana*, *Grosseria vignei*, *Physalis micrantha*, and *Stachytarpheta angustifolia.*

## 2. Methods

### 2.1. Plant Material Preparation

#### 2.1.1. Collection and Authentication of Plant Materials

The plant samples, *Celtis africana*, *Grosseria vignei*, *Physalis micrantha*, and *Stachytarpheta angustifolia*, were collected from Kwahu Asakraka, (6°38′05.2″N 0°41′20.6″W) in the Eastern Region of Ghana. Sample collection was done between July and September 2018 with the help of a plant botanist, Mr. Clifford Asare of the Department of Herbal medicine, Faculty of Pharmacy and Pharmaceutical Sciences at the Kwame Nkrumah University of Science and Technology (KNUST). After collection, foreign materials were removed from the plant materials and washed under running water. These were then allowed to dry in air at ambient temperatures under a shade for up to 30 days. The dried plant material was cut into small pieces and milled into a course powder. Plant part used, families, local names, and indications of the selected plants used for the study have been described in Table 1.

#### 2.1.2. Extraction

To 100 g of the plant material, 500 mL of 99% ethanol was used for Soxhlet extraction for a minimum duration of 4 hours. The extract was then filtered to separate any residue from the menstruum. The filtrate was then concentrated *in vacuo* (Cole Parmer Rotary Evaporator N-1110, China) to dryness and transferred into screw-capped amber vials and stored below 4°C until it was ready for use.

### 2.2. Phytochemical Screening

The presence or absence of various phytochemicals—flavonoids, alkaloids, tannins, sterols, glycosides, and coumarins—were tested in the plant extracts by following standard procedures [19, 20].

### 2.3. *In vitro* Antiplasmodial Assay

For this assay, a chloroquine-sensitive *Plasmodium falciparum* 3D7 strain was used. The *Plasmodium* parasite was added to freshly prepared human erythrocytes (O^+^) that were suspended in complete RPMI-1640 parasite medium enhanced with 10% normal human serum and then gassed with a gas mixture (2% O_2_, 5.5% CO_2_, and 92.5% N_2_—Air Liquide, UK). The parasite culture was incubated (RS Biotech, USA) at 37°C and maintained daily by changing the media. Stock solutions of the plant extracts were made in 70% ethanol. Dilution of the stock solutions yielded working solutions with concentrations between 0.5 *μ*g/mL and 600 *μ*g/mL which was used for the assay. Synchronization of the *Plasmodium* culture was made with 5% sorbitol to obtain the ring stage of the parasite, which was used in the assay. Working solutions of the synchronized parasites were prepared in a complete medium. In a flat-bottom 96-well microtiter plate containing 50  *μ*L of plant extract, 1.5% cell suspension of parasitized erythrocytes (about 0.9–1.5% parasitemia) was added. Artemisinin was used as a standard drug and treated similarly to the extracts. Solvent controls were also included. Microtiter plates were gassed in a desiccator with a gas mixture of 2% O_2_, 5.5% CO_2_, and 92.5% N_2_. The reaction mixture was incubated at 37°C for 72 hours. Upon assay termination, thin smears were made on glass slides from each duplicated well, fixed in methanol, and then dried. The slides were stained with Giemsa stain (Fluka Chemicals, UK), washed, dried, and examined using 100x magnification oil immersion objective lens of light microscope (Leica 1349522X, USA). The number of infected RBCs in a given grid area was counted. For each slide, a minimum of 1000 RBCs were counted [19, 21, 22]. The percentage of parasitemia was computed using the following expression:

(1) $$\begin{array}{c} \text{\% parasitemia}=\frac{\text{number of infected RBCs}}{\text{total number of RBCs}}\times 100. \end{array}$$

Dose-response curves were plotted and used to compute half-maximal inhibitory concentrations (IC_50_). Microsoft Excel and GraphPad Prism were used for all data and statistical analysis. All experiments were performed in triplicate.

### 2.4. Antioxidant Assays

#### 2.4.1. DPPH Radical Scavenging Assay

The 2, 2-diphenyl-1-picrylhydrazyl (DPPH) free radical scavenging method was used in estimating the radical scavenging activity of the extracts. A 0.1 mM solution of DPPH in methanol was prepared and introduced into wells in a microtiter plate. To each of the wells, 100 *μ*L of each varying concentration of extract prepared in methanol was added and mixed. The mixture was then incubated in the dark for 30 min. Absorbance was taken at 517 nm (Multimode Microplate Reader, BioTek Synergy H1, Germany). A methanol control blank was made and ascorbic acid was used as a standard drug. All tests were conducted as triplicates on the microtiter plate. Percentage of DPPH radical scavenged was calculated as follows:

(2) $$\begin{array}{c} \%\text{ DPPH radical scavenged}=\left(\frac{\text{Ac}-\text{As}}{\text{Ac}}\right)\times 100, \end{array}$$

where *Ac* represents the absorbance of control and *As* represents the absorbance of each test sample. The DPPH radical scavenging activity was reported as IC_50_'s from a dose-response curve which was obtained by plotting % DPPH radical scavenged against extract concentration [23].

#### 2.4.2. Total Antioxidant Capacity

The phosphomolybdenum (PM) method was used to estimate the total antioxidant capacity. The PM reagent was prepared by adding 2 mL each of 0.6 M sulfuric acid, 28 mM sodium phosphate, and 4 mM ammonium molybdate to 94 mL of distilled water. To 0.1 mL of each sample solution, (various concentrations) 1 mL of the PM reagent was added. The mixture was then capped and incubated in a water bath at 90°C for 90 min. After cooling to room temperature, absorbance was measured at 695 nm (Shimadzu UV-1280 UV-Vis Spectrophotometer, Japan) against a blank. The blank solution contained 1 ml of the PM reagent solution and 0.1 of distilled water and incubated under the same conditions as the test samples. Ascorbic acid was used as standard and was used in the generation of a calibration curve. All tests were conducted in triplicate. The total antioxidant capacity was expressed in ascorbic acid equivalents (mg/g AAE) [23].

#### 2.4.3. Total Phenolic Content

Folin and Ciocalteu's method was used in the assessment of total phenolic content (TPC). Different concentrations of the extracts were made in methanol. The reaction mixture contained an aliquot of each sample (0.5 mL), 10% Folin–Ciocalteu's reagent (2.5 mL), and 7.5% sodium carbonate (2 mL). This was then made to stand for 30 min at room temperature before absorbance readings at 760 nm (Shimadzu UV-1280 UV-Vis Spectrophotometer, Japan). Gallic acid was used as a standard and was used in generating a calibration curve. All experiments were conducted in triplicate. The TPC was expressed in gallic acid equivalents (mg/g GAE) [23].

### 2.5. Toxicological Evaluation

#### 2.5.1. Acute Toxicity

Experiments were conducted according to the Organization for Economic Cooperation and Development (OECD) guidelines for testing of chemicals' acute oral toxicity [24]. The acute toxicity of the extracts was assessed in female Balb/C mice, aged 8–10 weeks (20–40 g body weight). Five groups of 5 animals were used in the study. Prior to the experiment, all animals in the various groups were kept in their respective cages for 7 days for acclimatization under a 12 hour dark/light cycle. Food and water were freely available. Groups 1 to 4 received extracts whereas group 5 received vehicle only (saline). Animals in groups 1–4 received plant extract at a fixed dose of 2000 mg/kg. The animals were observed immediately after extract or saline administration; at 30 minutes, 60 minutes, 4 hours, and 24 hours after administration; and then once a day for the next 14 days for signs of behavioral changes (changes in hair, eyes, skin, mucous membrane, salivation, lethargy, diarrhea, and sleep) or mortality. Feed and water consumption were monitored. Body weights were recorded on days 7 and 14 [25].

#### 2.5.2. Serum Analysis

Mice were anesthetized on day 15 and blood samples were collected from their chest cavity into gel tubes with no anticoagulants. Blood samples were then centrifuged at 3000 rpm for 10 minutes to obtain the serum. The obtained serum was stored at 4°C for the evaluation of biochemical parameters—aspartate transaminase, alanine transaminase, alkaline phosphatase, total bilirubin, direct bilirubin, indirect bilirubin, albumin, globulin, total protein, creatinine, and urea. The evaluation of the biochemical parameters for kidney and liver functions was done on an Automated Clinical Chemistry Analyzer (Le Scientific Medfuture LCC, USA) using commercially available kits (Anamol Laboratories Pvt. Ltd., India) [19].

## 3. Results

Four plants commonly used in folkloric medicine in Ghana for the treatment of malaria and malaria-like symptoms were selected for this study. The stem bark, leaves, and twigs of the plants are mostly used in herbal medicine and so these parts were employed here. Table 1 provides the botanical name, family, local name, plant part used, and local indication of selected plants used in the study. Extraction of plant materials by Soxhlet gave the products in appreciable yields, as seen in Table 2. The yield was in the order *Celtis africana* > *Grosseria vignei* > *Stachytarpheta angustifolia* > *Physalis micrantha*. The selected plants were evaluated for the presence or absence of 6 common phytochemicals: flavonoids, alkaloids, tannins, sterols, glycosides, and coumarins, and the results are presented in Table 3. All selected plants tested positive for the presence of tannins whereas none tested positive for the presence of alkaloids. *Celtis africana* did not give a positive test for flavonoids whereas *Stachytarpheta angustifolia* is the only plant that gave a positive result for the presence of glycosides.

Using the classification standards of Jonville et al. [26], the antiplasmodial activity against *Plasmodium falciparum* 3D7 strain was classified (Table 2). *Physalis micrantha* had the best antiplasmodial activity with an IC_50_ value of 3.51 ± 0.19 *µ*g/mL. *Celtis africana* had a moderate antiplasmodial activity with an IC_50_ value of 29.05 ± 1.29 *µ*g/mL. *Grosseria vignei* and *Stachytarpheta angustifolia* were classified as inactive with IC_50_ values greater than 50 *µ*g/mL. The standard drug, artemisinin, recorded an IC_50_ of 0.014 ± 0.001 *µ*g/mL.  Table 4 shows the results for the total antioxidant capacity, radical scavenging ability, and total phenolic content of the plant extracts. The total antioxidant capacities of the selected plant extracts were within the ranges of 800 to 840 mg/g AAE, indicating very good activities. *Stachytarpheta angustifolia* extract exhibited the best total antioxidant capacity of about 836.8 ± 5.57 mg/g AAE. This was followed by extracts of *Celtis africana* and *Grosseria vignei.* Extract of *Physalis micrantha* exhibited the lowest TAC. In the DPPH radical scavenging activity, IC_50_ values obtained ranged between 395 and 906 *µ*g/mL for 4 plant extracts. *Physalis micrantha* had the best DPPH scavenging activity with an IC_50_ value of 395 ± 7.90 *µ*g/mL. In comparison, the standard ascorbic acid standard had an IC_50_ of 36.34 ± 1.06 *µ*g/mL. The order of activity in the DPPH radical scavenging assay was *Physalis micrantha* > *Celtis africana* > *Stachytarpheta angustifolia* > *Grosseria vignei*. There was a direct correlation between antiplasmodial activity and antioxidant activity, with the same order of activity of plant extracts observed in both assays. Due to the role of phenolic compounds as potential antioxidants, the total phenolic content (TPC) of the extracts was also investigated. TPC values ranged between 15 and 109 mg/g GAE. *Grosseria vignei* recorded the highest TPC, with *Physalis micrantha* having the lowest. There was no direct correlation between DPPH activity and TPC in this study.

**Figure 1:**
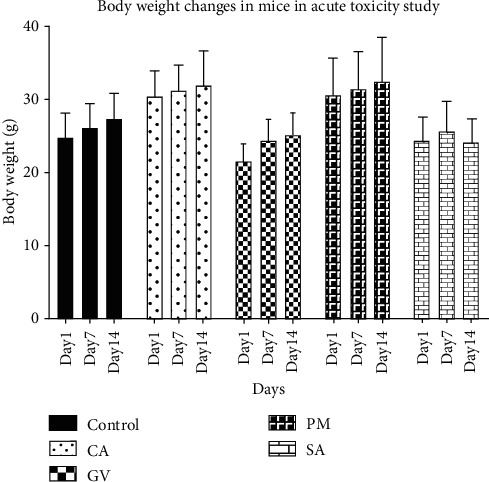
Body weight changes in mice in acute toxicity study. Each bar represents mean ± SEM (*n* = 5). CA: *Celtis africana*, GV: *Grosseria vignei*, PM: *Physalis micrantha*, SA: *Stachytarpheta angustifolia*.

In the acute toxicity test, mice were dosed with a fixed amount of extract or saline and the mice were studied over a 14-day period. The clinical appearance of all mice in the various groups, including the control group, did not show any treatment-related adverse effects. No deaths were recorded in any of the groups and all morphological and behavioral observations were normal. Body weight monitoring over the period of the study revealed a gradual increase in weight in mice as seen in Figure 1. In the control group, an average weight increase of 9.38% was observed after 14 days. Groups that received *Physalis micrantha*, *Celtis africana*, *Stachytarpheta angustifolia*, and *Grosseria vignei* recorded body weight increases of 6.44, 5.43, −1.15, and 14.73%, respectively, between days 1 and 14. The half-maximal lethal dose (LD_50_) for all extracts was estimated to be over 2000 mg/kg.

**Figure 2:**
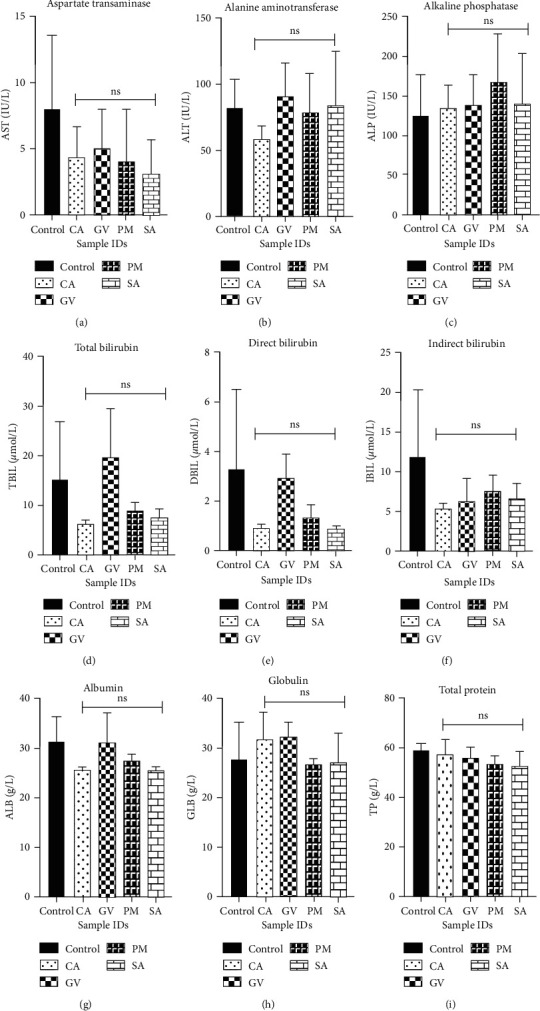
Effect of selected plant extracts on liver function profiles (*n* = 3). CA: *Celtis africana*, GV: *Grosseria vignei*, PM: *Physalis micrantha*, SA: *Stachytarpheta angustifolia.* (a) AST: aspartate transaminase, (b) ALT: alanine transaminase, (c) ALP: alkaline phosphatase, (d) TBIL: total bilirubin, (e) DBIL: direct bilirubin, (f) IBIL: indirect bilirubin, (g) ALB: albumin, (h) GLB; globulin, and (i) TP: total protein. Each bar represents mean ± SEM (*n* = 3). *p* < 0.05 was considered to be statistically significant (one-way analysis of variance (ANOVA) followed by Dunnett's *post hoc* test).

**Figure 3:**
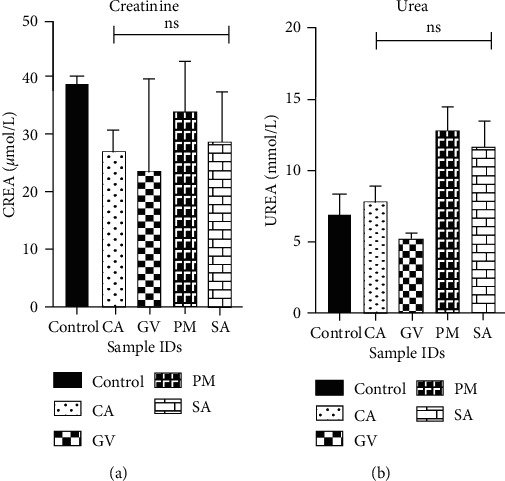
Effect of selected plant extracts on kidney function profile (*n* = 3). CA: *Celtis africana*, GV: *Grosseria vignei*, PM: *Physalis micrantha*, SA: *Stachytarpheta angustifolia.* (a) CREA: creatinine and (b) UREA: urea. Each bar represents mean ± SEM (*n* = 3). *p* < 0.05 was considered to be statistically significant (one-way analysis of variance (ANOVA) followed by Dunnett's *post hoc* test).

The activities of various enzymes in blood drawn on day 15 from animals in the various treatment groups were used as markers for the hepatic and renal status of animals after extract treatment. For hepatic function, activities that corresponded to aspartate transaminase, alanine transaminase, alkaline phosphatase, total bilirubin, direct bilirubin, indirect bilirubin, albumin, globulin, and total protein were determined (Figure 2). The levels of these markers in animals in the control group and the extract-treatment groups showed no significant statistical differences (*p* > 0.05). For kidney function, the levels of urea and creatinine were used as markers and the results are shown in Figure 3. Similar to the results in the liver function test, no significant differences were observed between animals in the extract-treatment groups and the control groups.

## 4. Discussion

The four medicinal plants used in this study are frequently used in various herbal preparations in Ghana. Ethanolic extracts of the various plants were obtained in yields comparable to other plant extraction experiments reported elsewhere [25, 27]. Reports on several bioactivities of extracts from these plants are ubiquitous in the literature. These bioactivities include antimicrobial, anti-inflammatory, antioxidant, antidiabetic, neuropharmacological, laxative, antidiarrheal, gut, and immunomodulatory [27–33]. Despite widespread use in medicinal herbal formulations, very little information exists on the biological activities of extracts of *Physalis micrantha.* Extracts of *Stachytarpheta angustifolia* have been reported to possess antibacterial, antioxidant, immunomodulatory, and antidiabetic properties [30, 34]. *Grosseria vignei* has been shown to possess antioxidant and anti-inflammatory activities [29]. Interestingly, no detailed reports of the antiplasmodial activities of these plants exist in the literature, with the exception of *Celtis africana*. Crude solvent extracts of the leaf and stem of *Celtis africana* possess antiplasmodial activity against the *Plasmodium falciparum* parasite with an IC_50_ value of about 30 *µ*g/mL [35, 36]. It has also been shown that the stem bark extract contains good antioxidant and anti-inflammatory compounds [27].

Various classes of phytochemicals have been shown to possess interesting antiplasmodial properties. These include alkaloids, terpenes, flavonoids, limonoids, chalcones, and coumarins [37]. Flavonoids and tannins were present in all plant extracts studied (Table 3) and these phytochemicals probably play key roles in the biological activity of these plant extracts. In the antiplasmodial activity test, extracts of *Physalis micrantha* and *Celtis africana* were found to have high and moderate activity against the *Plasmodium falciparum* parasite with IC_50_ values of 3.51 and 29.05 ± 1.29 *µ*g/mL, respectively (Table 2). The IC_50_ value obtained in this work for *Celtis africana* is very similar to other reports on its antiplasmodial activity [35, 36]. These IC_50_ values are an indication of good antiplasmodial activity of the two plant extracts and partly provide scientific backing for their use in local herbal preparations for malaria treatment. The low IC_50_ values recorded for extracts of *Physalis micrantha* and *Celtis africana* are probably as a result of the synergistic action of one or more of the phytochemical constituents present in these plant extracts, as reported by other works [38, 39]. Flavonoids, in general, have been reported as potent secondary metabolites of plants possessing broad spectrum biological activities [37, 40]. *Grosseria vignei* and *Stachytarpheta angustifolia* were classified as being inactive in the antiplasmodial assay due to their high IC_50_ values (>50 *µ*g/mL). For this study, the ring stage of the *Plasmodium falciparum* parasite was used. It is possible that *Grosseria vignei* and *Stachytarpheta angustifolia* extracts have little to no effect on the parasite at this stage but may inhibit other stages of the parasite. It is also known that some extracts without *in vitro* antiplasmodial activities may possess remarkable *in vivo activity*—via stimulating of immune responses. Extracts of *Markhamia lutea* were active against *Plasmodium berghei in vivo* even though they were inactive in *in vitro* assays [32]. Some of the extracts used in this study may fall within this category. Additionally, most herbal preparations are a combination of two or more plants [18]. Synergistic action is thus expected in such situations.

Reactive oxygen and nitrogen species are major factors in the induction of oxidative stress in cells. These species have been suggested to play important roles in systemic complications that occur during malaria infection. During malaria infection, hydroxyl radicals are produced in the liver and these radicals contribute significantly to the induction of cellular oxidative stress. It has also been shown that normal red blood cells produce about 50% fewer hydroxyl radicals and hydrogen peroxide than *Plasmodium falciparum*-infected red blood cells [41]. Additionally, FP presence in the host cell contributes to oxidative imbalance [13]. Together, this set of data points to an important role of reactive species during malaria infection. This necessitated the investigation of the antioxidant activities of the plant extracts. All extracts displayed very high activities in the phosphomolybdenum test, with total antioxidant capacities greater than 800 mg/g AAE. In the DPPH radical scavenging assay, the extracts showed good activities as well. There was a good agreement between the DPPH radical scavenging results and the antiplasmodial assay results. Extracts of *Physalis micrantha* displayed the best DPPH scavenging ability. Interestingly, this same extract showed very high antiplasmodial activity. It is thus possible that the extract exerts its antiplasmodial activity by contributing to oxidative balance in an infected cell. Phenolic compounds have been suggested as an important class that mops up free radicals [42]. However, there is no agreement about the correlation between antioxidant activity and TPC. In this study, no direct correlation was observed between TPC and antioxidant activities.

In the acute toxicity studies, no mortality was recorded in any of the extract-treatment groups at the maximum dose of 2000 mg/kg of extract administered. A single high dose is recommended for the evaluation of acute toxicity [24]. All behavioral and morphological observations made in all treatment and control groups were also normal. Therefore, the LD_50_ (median lethal dose) of all extracts was estimated to be greater than 2000 mg/kg [24], and as such, all extracts are rated as safe up to this dosage level. Change in body weight of experimental animals is frequently used as an index of toxicity, as it is simple yet very sensitive [43, 44]. An increase in body weight was observed for animals in the control group as well as treatment groups that received *Physalis micrantha*, *Celtis africana*, and *Grosseria vignei*. Animals in the group that received *Stachytarpheta angustifolia* extract experienced a marginal reduction in body weight over the 14-day experimental period. However, there was no significant change (*p* > 0.05) in body weight in animals in that group from days 1 to 14.

Medicinal plants with a history of long usage in the treatment of diseases are usually perceived to be safe. Contrary to this notion, there have been a number of reports in the recent literature that reports on adverse effects of plant extracts on the liver and kidneys [17, 45]. Metabolic defects and injuries to these organs can be deduced from changes in the levels and activities of key biomarkers. Aspartate transaminase, alanine transaminase, alkaline phosphatase, total bilirubin, direct bilirubin, indirect bilirubin, albumin, globulin, and total protein are biomarkers of liver function and changes in their levels and/or activities are indicative of toxicity. In this study, no significant differences (*p* > 0.05) were observed in the levels of the markers in the control group and the extract-treatment groups. In the kidney function test, the levels of creatinine and urea were used to evaluate toxicity to the kidney. Similar to results in the liver function test, no significant differences were observed between animals that received the vehicle and those that received the extracts. Thus, it can be deduced that the extracts at 2000 mg/kg posed no adverse effect to the proper functioning of the liver and kidney and are safe at doses at or below this concentration.

## 5. Conclusion

Extracts of *Physalis micrantha* and *Celtis africana* possessed very good antiplasmodial activities. On the other hand, extracts of *Grosseria vignei* and *Stachytarpheta angustifolia* displayed no activity towards the *Plasmodium falciparum* strain used in the study. All extracts showed great potential in mopping up reactive species, as evidenced by their strong antioxidant activities. All extracts were safe at doses at/or below 2000 mg/kg. The results of this study provide important information about the antiplasmodial and antioxidant activities of the plant extracts and partly provide some scientific basis for their use in folkloric medicine.

## Figures and Tables

**Table 1:** Botanical name, family, local name, part used, and local indication of selected plants.

Botanical name	Family	Local name (Twi)	Plant part used (voucher specimen number)	Local indications
*Celtis africana*	Cannabaceae	Essa	Stem bark (KNUST/HMI/2021/SB011)	Fever/headache/sore
*Grosseria vignei*	Euphorbiaceae	Dubrafor	Leaves (KNUST/HMI/2021/L006)T wigs (KNUST/ HMI/2021/T001)	Wound healing/fever
*Physalis micrantha*	Colanaceae	Toto nini	Leaves (KNUST/HMI/2021/L007)T wigs (KNUST/ HMI/2021/T002)	Malaria/painkiller/epilepsy
*Stachytarpheta angustifolia*	Verbenaceae	Kwankurahu	Leaves (KNUST/HMI/2021/L008)T wigs (KNUST/ HMI/2021/T003)	Venereal diseases/dropsy/fever

**Table 2:** Extract yield and *in vitro* antiplasmodial activity of selected plants against *Plasmodium falciparum* 3D7 strain.

Plant extract	Extract yield^*∗*^ (%)	Antiplasmodial activity—IC_50_ (*μ*g/ml)	Classification^*∗∗*^
*Celtis africana*	15.2	29.05 ± 1.29	Moderate
*Grosseria vignei*	7.3	>50	Inactive
*Physalis micrantha*	5.4	3.51 ± 0.19	High
*Stachytarpheta angustifolia*	5.7	>50	Inactive
Artemisinin	NA	0.014 ± 0.001	NA

NA—not applicable. ^*∗*^Extract yield percentage based on dry pulverized plant material. ^*∗∗*^Classification of antiplasmodial activity based on [26]—high (IC_50_ < 5 *µ*g/mL), promising (5 < IC_50_ < 15 *µ*g/mL), moderate (15 < IC_50_ < 50 *µ*g/mL), and inactive (IC_50_ > 50 *µ*g/mL).

**Table 3:** Phytochemical composition of selected plants.

Plant extract	Flavonoids	Alkaloids	Tannins	Sterols	Glycosides	Coumarins
*Celtis africana*	+	−	++	−	−	+
*Grosseria vignei*	++	−	++	−	−	+
*Physalis micrantha*	+	−	++	−	−	−
*Stachytarpheta angustifolia*	++	−	++	++	+	−

(−)—not detected; (+)—present; (++)—strongly present.

**Table 4:** Antioxidant capacity, radical scavenging, and total phenolic content of selected plants.

Plant extract	Antioxidant capacity mg/g (AAE)	DPPH radical scavenging IC_50_ (*µ*g/mL)	Total phenolic content mg/g (GAE)
*Celtis africana*	835.59 ± 1.56	754 ± 4.87	98.7 ± 2.43
*Grosseria vignei*	825.10 ± 4.11	905.5 ± 2.66	108.5 ± 2.79
*Physalis micrantha*	800.9 ± 4.34	395 ± 7.90	15.26 ± 09.10
*Stachytarpheta angustifolia*	836.8 ± 5.57	765 ± 4.84	72.1 ± 1.47
Ascorbic acid	NA	36.34 ± 1.06	NA

NA—not applicable.

## Data Availability

All data generated or analyzed during this study are included in this published article.
